# COVID-19 and the Kidney: A Worrisome Scenario of Acute and Chronic Consequences

**DOI:** 10.3390/jcm10050900

**Published:** 2021-02-25

**Authors:** Luis Sanchez-Russo, Marzuq Billah, Jorge Chancay, Judy Hindi, Paolo Cravedi

**Affiliations:** Department of Medicine, Icahn School of Medicine at Mount Sinai, New York, NY 10029, USA; Luis.SanchezRusso@mountsinai.org (L.S.-R.); Marzuq.Billah@mountsinai.org (M.B.); Jorge.Chancay@mountsinai.org (J.C.); Judy.Hindi@mountsinai.org (J.H.)

**Keywords:** AKI, CKD, COVID-19, CG, CoV-AKI, ATI, ESKD

## Abstract

Acute kidney injury (AKI) is a common finding in patients with coronavirus disease 2019 (COVID-19) and has been associated with higher rates of death when compared to COVID-19 patients without kidney injury. Whereas the definitive pathogenesis of COVID-19-related AKI (CoV-AKI) is not clear, histopathologic evidence seems to point at multiple etiologies for the disease, including indirect and direct viral kidney injury. The high incidence of CoV-AKI, along with the aggressive clinical presentation of this entity, have increased the demands for kidney replacement therapies, rapidly overwhelming the supplies of healthcare systems even in major tertiary care centers. As a result, nephrologists have come up with alternatives to maximize the efficiency of treatments and have developed non-conventional therapeutic alternatives such as the implementation of acute peritoneal dialysis for critically ill patients. The long-term implications of CoV-AKI are yet unknown, though early studies suggest that around one third of the patients who survive will remain dependent on kidney replacement therapy. Nephrologists and healthcare workers need to be familiar with the clinical presentation and therapeutic challenges of CoV-AKI in order to develop strategies to mitigate the burden of the disease for patients, and for services providing kidney replacement therapies.

## 1. Introduction

It is estimated that the number of fatal cases of coronavirus disease 2019 (COVID-19) will near 500,000 in the United States (US) by the time this review is published [1]. Calculations report that more than 2.5 million person-years of life have been lost in the US alone [2]. In addition to fatality, the potential health implications of acute and chronic end-organ damage resulting from direct or indirect injury during the early infection phase represent another major consequence of the severe acute respiratory syndrome coronavirus 2 (SARS-CoV-2) pandemic. Data indicate a strong association between COVID-19 and kidney injury, and though causality is less clear, this strong association could lead to a surge in the prevalence of chronic kidney disease (CKD) from COVID-19-related acute kidney injury (AKI). Therefore, understanding the pathophysiology of COVID-19 kidney injury, response to therapy, and prognosis is imperative for clinicians not only to build competency in the treatment of the disease, but also to build projections of the reminiscence of kidney disease once the pandemic is over. Herein, we review data on COVID-19 epidemiology, pathogenesis, and the therapeutic challenges of patients with AKI related to COVID-19 (CoV-AKI). Though this article includes data coming from the experience of groups from all over the world, we focus mainly on the data obtained from the experience in centers in the US, one of the current epicenters of the COVID-19 pandemic.

### Sars-CoV-2

In December 2019, a novel strain of Coronavirus was identified as the causative agent on a cluster of patients presenting with severe pneumonia in Wuhan, China. Airway epithelial cells from affected individuals were used to isolate the responsible coronavirus named 2019-nCov or SARS-CoV-2, different from Middle East respiratory syndrome coronavirus (MERS-CoV), and severe acute respiratory syndrome coronavirus (SARS-CoV) [3]. Since the report of the initial cases in Wuhan, the spread of the virus has caused over ninety million cases and over two million deaths worldwide [4].

Coronaviruses are a family of RNA viruses that belong to the Nidovirus superfamily [5]. The zoonotic origin in the outbreak of other strains of coronavirus such as the aforementioned SARS-CoV and MERS-CoV featured as an archetype for the hypothesis of animal to human transmission of this new virus. To date, multiple relatives of the novel coronavirus have been isolated in bats and pangolins, some of which share above 95% of the genomic sequence with SARS-COV-2 [6]. However, the small genomic differences may still be the result of decades on viral evolution; thus, to date, the reservoir host of the virus has not been found, and the animal to human transmission model has not been formally proven [7].

COVID-19, the disease associated with SARS-CoV-2, has a broad-spectrum presentation ranging from individuals who are asymptomatic or presenting with mild symptoms such as fever and myalgia to severe cases characterized by acute respiratory distress syndrome (ARDS), hypercoagulability, and multiple organ failure. AKI has been among the dominant extra pulmonary finding in patients with moderate to severe COVID-19 despite negligible incidence in initial reports [8].

## 2. Epidemiology of CoV-AKI

Incidence of AKI in patients with COVID-19 varies greatly among reports from different geographic locations [9,10,11,12,13,14]. Heterogeneity in the definition of AKI complicates this discussion even further. Five recently published meta-analyses including, overall, more than 20,000 patients, describe an incidence of AKI ranging from 4.5% to 18% in hospitalized patients with COVID-19 [15,16,17,18,19]. However, these reports have several issues to exhibit a plausible worldwide incidence of AKI within COVID-19-infected patients. Problems include over-representation of Chinese populations, wide range of disease severity within same cohorts, virtually no representation of European, Latin American or African countries, and the same cohorts being used within these different meta-analyses [15,16,17,18,19].

Initial descriptions from countries first affected in Asia reported a lower incidence of AKI when compared to data obtained from Europe and North America [12,14,19,20]. In two single-center reports from China, AKI among hospitalized patients is described to be between 4.5% and 7%, with higher incidences within cohorts including critically ill patients [21,22,23,24,25,26]. This is in contrast with data available from the United States. For example, a multicenter report with a cohort of 5700 hospitalized patients across New York State reported an incidence of AKI of 22%, with 14% of those patients requiring stay in the intensive care unit (ICU) [27]. Other reports from large centers in the US show higher incidence of AKI ranging from 24% to 46% among patients with COVID-19 who were admitted to the ICU [9,27,28,29,30]. A summary of incidence reports is available in Table 1.

Information regarding kidney complications of COVID-19 from Africa and Latin America is scarce. A large report of two centers in South Africa described an AKI incidence of 33.9% within hospitalized patients with COVID-19. Of these patients, 14% required ICU stay [31]. Furthermore, of the patients with AKI, 8.6% required kidney replacement therapy (KRT). Differently, a cohort from Casablanca, Morocco showed a lower incidence of AKI within patients requiring ICU stay, reporting an AKI incidence of 17.8% [32]. Preliminary, non-published data from the Latin American Society of Nephrology and Hypertension (SLANH) report that 50.3% of 393 hospitalized patients with CoV-AKI required KRT, the highest reported until now [33]. This is in contrast with a multicenter observational study across ten ICUs from Mexico which reported that 14.6% of patients hospitalized in critical care units required KRT; the incidence of AKI was not reported [34].

Multiple risk factors have been associated with developing AKI in patients with COVID-19. Similarly to non CoV-AKI, significant risk factors include respiratory failure requiring mechanical ventilation and need for vasoactive agents [9,21,29,35,36]. A registry of 5449 patients reported that 89% of the patients requiring mechanical ventilation developed AKI [9]. In the US, an observational study on 575 patients admitted to the ICU with COVID-19 showed that 61% of the patients that required vasoactive medications had AKI [35]. Other risk factors described for developing AKI associated to COVID-19 include older age, male gender, comorbidities such as diabetes mellitus (DM), hypertension (HTN), obesity or cardiovascular disease, being Black and being Hispanic [18,19,21,26,27,36].

The reported incidence of AKI within patients with COVID-19 that required KRT in US is between 3.2% and 20% [18,20,26,27,35,36,37,38]. In a retrospective observational study, the incidence of severe AKI requiring KRT was higher among COVID-19 patients than in similar patients with AKI secondary to etiologies different from COVID-19 (4.9% vs. 1.6%) [36]. Limitations of this study include the use of a historic cohort as the comparison group.

### 2.1. Mortality

AKI in COVID-19 patients is associated with higher mortality, but whether AKI is an independent risk factor for mortality or whether this association is confounded by the severity of the infection is still unclear. Overall, patients infected with COVID-19 who developed AKI had longer hospitalizations, were more likely to be admitted to an ICU, and had a higher mortality [9,11,21,26,29,36,37,38]. Moreover, mortality was directly proportional to the severity of AKI [9,21,26]. A large retrospective cohort study compared outcomes among patients with and without AKI who were admitted to a network of hospitals in metropolitan New York [39]. In-hospital mortality for COVID-19 patients with AKI was 46.4%, compared to 7.3% in those who did not develop AKI. The authors reported adjusted and unadjusted hazard ratios for patients who required KRT and those who did not. Independent of the adjustment model used, the hazard ratio for in-hospital death among patients who required KRT for AKI was two times higher compared to patients with AKI who did not require KRT. Unsurprisingly, the rates of ICU and mechanical ventilation where higher in the AKI group (6.3% and 2.4% vs. 45.2% and 40.8% for AKI stage 1–3 not receiving KRT, and 92.3% and 91.5% for those receiving KRT).

### 2.2. Kidney Recovery

A large retrospective study in New York City reported that the rate of kidney recovery among COVID-19 survivors who did not require KRT was 74.1%, compared to 66.7% of those who required KRT [39]. The group defined recovery of kidney function as a decline of 33% in serum creatinine at discharge compared to peak creatinine during admission, and no longer needing dialysis at discharge for those who required KRT. Similarly, another multicenter study that included a cohort of 3099 critically ill adults with COVID-19 identified that 34% of survivors who required KRT remained dependent on KRT on discharge [30]. Further studies are still needed to look at this patient population longitudinally after discharge. As of now, long-term kidney prognosis is unknown; however, it may be safe to speculate that prognosis will be associated to etiology, and that those with features of collapsing glomerulopathy (CG) and thrombosis may have a higher risk of being KRT-dependent compared to those with other types of injury, such as acute tubular injury (ATI).

## 3. Pathogenic Mechanisms and Histopathology Findings of COVID-19-Related AKI

Different series of kidney biopsies and autopsy reports from patients with COVID-19-related AKI support the concept that kidney injury happens as the result of multiple causes (Table 2) [8]. Acute tubular injury (ATI), inflammatory infiltrates in the interstitial space, endothelial damage, and collapsing glomerulopathy are among the features frequently seen in the kidney biopsy series available for this patient population [40,41,42,43].

In general, the pathogenesis of CoV-AKI includes two major causes: (1) indirect injury resulting from the inflammatory and hemodynamic changes during COVID-19 and (2) direct injury induced to the organ by SARS-CoV-2.

### 3.1. Indirect Mechanisms of SARS-CoV-2 Kidney Injury

An uncontrolled inflammatory state induced by SARS-COV-2 infection and the resultant hemodynamic instability are likely to account for the majority of the cases of CoV-AKI. It is not coincidental then that most of the kidney biopsy series available for COVID-19 patients report variable degrees of classic ATI. AKI in COVID-19 may then follow similar pathogenic models to sepsis-induced AKI.

A histopathological analysis on 26 autopsies in patients with COVID-19 described ATI in the proximal convoluted tubule (PCT) based on loss of brush border, vacuolar degeneration, and dilation of tubular lumen with cellular debris [44]. A few of the cases also had frank necrosis with detached epithelium in the tubules. Light microscopy showed glomerular loops and peritubular capillaries with diffuse erythrocyte aggregation and obstruction. Their study showed that ATI severity ranged from mild to severe. Additionally, the authors described that 16 of the 26 patients were hypotensive or require vasopressor support. ATI was then likely the result of ischemia secondary to hypoperfusion.

Cytokine induced kidney injury is another proposed mechanism to the pathogenies of CoV-AKI. Several pro-inflammatory interleukins (IL), interferon (IFN), colony stimulating factors, tumoral necrosis factors (TNF), and chemokines are all potential mediators of AKI. The term cytokine storm was originally used to describe a syndrome of immune dysregulation that developed after chimeric antigen receptor (CAR) T-cell therapy [46]. Cytokine storm can lead to acute lung injury, acute respiratory distress syndrome (ARDS), endothelial cell apoptosis, capillary leak syndrome, and broad systemic inflammation [47,48]. Macrophage activating syndrome has been implicated in this maladaptive cycle leading to continued production of cytokines [49]. Using tubuloreticular inclusions as a biopsy marker of endogenous or exogenous IFN, association has been made between COVID-19-related AKI and cytokine-mediated injury [41,50]. Although capable of resulting in end-organ damage, it is not entirely clear to what extent circulating cytokines contribute to the pathogenesis of CoV-AKI, bearing in mind that circulating levels of cytokines like interleukin-6 (IL-6) are considerably lower in patients with COVID-19 when compared to those seen after CAR T-cell therapy or sepsis. Nonetheless, the reported incidence of AKI in COVID-19 seems above the expected for sepsis [8]. Though variable, higher levels of certain cytokines, especially IL-6, have been correlated with AKI and mortality in certain patient populations, such as those with kidney transplant [51,52].

Organ crosstalk may also play a role on the development of kidney injury in patients with COVID-19 [53]. Several studies demonstrate the relationship between hypoxia, hypercarbia, and ARDS-related inflammatory cascade (IL-1B, IL-6, IL-8, and TNF-α) on kidney function. The consequences include decreased kidney blood flow, reduced glomerular filtration rate, and reduction in kidney cell viability. Koyner and Murray discuss in detail the relationship between positive pressure ventilation and kidney injury [54]. They describe effects in animal studies that include diminished cardiac output, sympathetic nervous system stimulation, increased arginine vasopressin, suppression of atrial natriuretic peptide, and decreased kidney blood flow. Lung injury from AKI involves many pathways, including the production of pro-inflammatory cytokines, neutrophil migration, T-cell activity, and pulmonary edema [55]. This effect is bidirectional and results in propagation of lung injury, as well. In light of this evidence, and in the setting of obvious pulmonary injury resulting from SARS-CoV-2, it is feasible to think that organ crosstalk may be credited for kidney injury to some extent.

### 3.2. Direct Mechanisms of SARS-CoV-2 Kidney Injury

SARS-CoV-2 uses the angiotensin converting enzyme 2 (ACE2) receptor to enter cells [6]. The virus has high tropism for respiratory tissue where it is capable of active replication and generation of secondary viremia, allowing an attack on other organs expressing ACE2 [56]. ACE2 expression in kidneys is approximately 100-fold greater than in the lung [57]. The CD147 spike protein has recently been described as a viral entry target as well, and it is highly expressed on proximal tubule epithelium and inflammatory cells [58]. Direct injury from SARS-CoV-2 to kidney cells along with predisposition of susceptible individuals such as those with high risk apolipoprotein L1 (APOL1) genotype may be factors accounting for patterns of glomerular and tubular injury seen repeatedly in kidney biopsies or autopsies from COVID-19 patients.

In vivo and ex vivo evidence of kidney tropism from SARS-CoV-2 is conflicting. Early evidence suggesting presence of SARS-CoV-2 in the kidneys came from Farkash et al. after adverting in the tubular epithelium particles which were morphologically identical to SARS-CoV-2 in the autopsy of a patient who died from COVID-19 [59]. However, other investigators attributed the morphologically similar particles to intracellular clathrin-coated vesicles and microvesicular bodies which are part of the endosomal pathway, as well as extracellular degenerate microvilli and extruded microvesicles from the abovementioned microvesicular bodies [60,61].

In a series of autopsies from COVID-positive patients, Puelles and collaborators detected viral load in kidney tissue using quantitative polymerase chain reaction (PCR) specific for SARS-CoV-2 in 13 out of 21 cases [62]. The same group also proceeded to do microdissection of glomerular and tubular fragments from an additional five patients, detecting SARS-CoV-2 viral load by PCR in all kidney compartments for three of these patients, with preferential targeting of glomerular cells. In this autopsy series, almost all patients who had available data had estimated glomerular filtration rate (eGFR) < 60 mL/min/1.73 m^2^ except for one patient. The investigators also found viral RNA and protein by using in situ hybridization (ISH) and indirect immunofluorescence (IF) in glomeruli and tubules. Kidney tropism was also corroborated by Braun et al. in a cohort of 63 patients [63]. In this postmortem analysis, SARS-CoV-2 RNA was detected in 60% of the patients. Moreover, the authors associated finding of SARS-CoV-2 RNA in the kidney with a decreased survival with a hazard ratio (HR) of 3.7 (95% CI 1.5–8.7; *p* = 0.003) [63].

On the other hand, a study performed by Kudose et al. utilized immunostains for viral spike and nucleocapsid phosphoproteins, ISH for viral RNA, and ultrastructure examination with electron microscopy to test for kidney tropism from SARS-CoV-2 in a series of 17 patients. Their techniques did not detect virions or viral RNA in the kidney [41]. One of the earliest case series to report CG in association with COVID-19 also failed to detect either SARS-CoV-2 RNA or any definitive viral particles in electron microscopy [45]. This biopsy series only had six patients, all presenting with CoV-AKI and proteinuria. A multicenter case series including 17 patients with CoV-AKI was not able to find evidence of direct infection of the virus to the kidney. The authors established three criteria based on the presence of viral particles, which in addition had to be present in the appropriate subcellular compartment compatible with known viral replication and trafficking pathway, plus the validation by immunohistochemistry or RNA ISH [43]. None of their cases met these criteria.

It is still unclear if the nephron is a direct target of SARS-CoV-2, and though several models may potentially be used to explain cellular damage to the kidney parenchyma, these are all speculative. If confirmed, the presence of the virus in kidney cells may induce a local immune response and complement activation, ultimately resulting in inflammation, cell lysis, and endothelial damage with thrombi formation [64]. Entry targets such as the ACE2 receptor and the CD147 protein may also exert roles in the pathogenic mechanisms for direct injury from the virus. CD147, for example, exerts a role in several kidney diseases through dysregulation of the cell cycle and inflammatory response [65]. CD147 induces kidney injury by inducing inflammation, ischemia, and loss of self-tolerance [66]. Overactivation of the angiotensin II pathway (Ang II) may also play a role in cellular damage by a different mechanism. ACE2 converts angiotensin II to angiotensin, which has an important effect in controlling inflammation, vasoconstriction, and thrombosis [65]. Upregulation of ACE and downregulation of ACE2 promote a proinflammatory and profibrotic state, which includes complement activation [67]. This may offer a plausible explanation for the damage of the tubular epithelia, as well as the increased incidence of thrombotic events.

## 4. Clinical Characteristics of CoV-AKI

A consequence of the variability in the pathogenesis of CoV-AKI is expecting similar variability in the clinical phenotype of this entity. Volume-responsive prerenal AKI may account for around 9% of the cases based on the report by Mohamed et al [35]. The same authors report in their series of 161 cases of CoV-AKI in the ICU that for a handful of patients (66%), the cause of AKI was settled to ischemic ATI. This numbers seem to be in concordance to other reports specially in regard to the high incidence of ATI. De novo glomerulopathies, especially CG, have been a recurrent finding on multiple case series [35,41,68].

### 4.1. Urine Abnormalities

Urinary abnormalities such as proteinuria and hematuria are very common in patients with SARS-CoV-AKI. Among 161 cases of CoV-AKI in New Orleans, de novo sub-nephrotic range proteinuria occurred among 39% of patients, and de novo nephrotic range proteinuria among 4% [35]. Proteinuria was also very prevalent in a retrospective study looking at 5449 patients admitted with COVID-19 across 13 academic and community hospitals in metropolitan New York. Urine studies were available for 646 out of 1993 patients with CoV-AKI, and proteinuria was found in 74% of the available samples. Urine protein quantified as 3 + was found in 12.1% [9]. The authors did not discern between de novo or preexisting proteinuria. Hematuria was a common finding in all the case series, while large or significant hematuria was uncommon.

In the abovementioned series, there are conflicting data in regard to urinary sodium. While Mohamed et al. describe low urinary sodium in only 38% of the cases, Hirsch reports low urine sodium in 65.6%. It is worth mentioning that Hirsch and collaborators used a higher cutoff for low urinary sodium (less than 35 meq/L), whereas Muner used 20 meq/L. This may potentially help to explain why there is a large discrepancy among the two studies.

Given the heterogeneity in the presentation of CoV-AKI, it is important to identify urine abnormalities in order to discern which patients may have AKI mediated by hemodynamic changes, and which patients may be presenting with glomerular injury and may benefit from a kidney biopsy. Limitations for the interpretation of existing data from urine studies in patients with COVID-19 AKI include the absence of serial urine measurements, especially in those patients with low urinary sodium, and the absence of separation of samples collected after indwelling catheter, which may have cause hematuria secondary to trauma.

### 4.2. Glomerular Injury

Susceptible individuals may be prone to the development of glomerular diseases during COVID-19. Collapsing glomerulopathy is the most common histopathologic finding in terms of glomerular diseases in patients with COVID-19. Patients may have different presentations ranging from isolated proteinuria in nephrotic ranges to AKI. While the pathogenesis is unclear, this feature is associated with other viral infections such as human immunodeficiency virus (HIV), Epstein–Barr, and parvovirus B-19 [69]. The proposed mechanisms of podocyte cytotoxicity include pro-inflammatory cytokine-mediated injury and/or direct viral injury [70]. There may be an association with APOL1 risk allele and COVID-19-related CG among Blacks which suggests that this group is more likely to develop APOL1-related kidney disease after exposure to COVID-19. This could also suggest that CG in COVID-19 is part of a second hit phenomenon in patients with APOL1 risk alleles [45,71]. Collapsing focal segmental glomerulosclerosis (FSGS) has been associated to therapeutic use of IFN-α, -β, and -γ [72]. Considering the robust interferon response in patients with COVID-19 at certain stages of the disease [73,74], it is then possible that IFN may be inducing podocyte injury in these patients.

### 4.3. CoV-AKI Associated with Hypercatabolic State

The immune response elicited by the viral infection has been associated with a hypercatabolic state similar to that seen in patients with rhabdomyolysis or tumor-lysis syndrome. Skeletal muscles loss has been linked to pro-inflammatory cytokines such as IL-6 and TNF-α [75,76], which are elevated during COVID-19 and correlate with disease severity [77]. A small case series from Mount Sinai Hospital in New York City reported nine critically ill patients with COVID-19-related AKI who had evidence of hyper catabolism characterized by high serum phosphorus and blood urea nitrogen (BUN), hyperkalemia, and hypocalcemia. None of the patients were known to have CKD. In this case series, creatine kinase (CK) levels were normal or slightly elevated. None of the patients required vasopressors, and none of them were receiving aggressive nutrition that could explain an increase exogenous load of phosphorus [78]. Another small study looked at the urea generation (URR) rate in eight patients who were started in acute peritoneal dialysis (PD) for CoV-AKI. URR was 2-fold higher for these patients when compared to stable patients receiving PD at home [79]. This trial was limited by the small number of patients and by the fact that stable end-stage kidney disease (ESKD) patients were used as comparison rather than patients with non CoV-AKI.

## 5. COVID-19 and Pre-Existing Kidney Disease

Patients with CKD, especially those with end-stage kidney disease receiving periodic KRT, are a vulnerable population for adverse outcomes from COVID-19 in light not only of their kidney condition, but also of comorbid conditions. Several studies in the US reported mortality up to 30% for this patient population [80].

### 5.1. COVID-19 in CKD Patients

To date, the largest study looking at the outcome of individuals with pre-existing kidney disease and COVID-19 comes from Flythe et al. on behalf of the STOP-COVID investigators [81]. This group reported near 50% mortality among those with pre-existing kidney disease compared to 35% mortality in individuals without pre-existing kidney disease. Even though this study was conducted on ICU patients, it provides evidence that patients with CKD are at increased risk when compared to individuals without pre-existing kidney disease. Moreover, dialysis patients had a shorter time from symptom onset to ICU admission.

### 5.2. COVID-19 in Patients Receiving Maintenance Dialysis

Patients receiving chronic in-center hemodialysis are at particularly high risk for SARS-CoV-2 exposure and infection given the inability to fully isolate [82]. A large study using the French registry of dialysis patients reported that the incidence of COVID-19 among patients receiving chronic dialysis was 3.3% among a total of 48,669 patients. The mortality among infected patients was around 21% [83]. In New York City, a retrospective study on 419 patients with ESKD who were admitted to academic and community hospitals reported a mortality rate of 31.7% with an odds ratio of 1.38 (95% confidence interval 1.12–1.7) when compared to patients who were admitted without the pre-existing diagnosis of ESKD [84]. Another study conducted within the Mount Sinai Health Care System in New York City looked at the outcomes of patients on maintenance dialysis who were hospitalized with COVID-19 [85]. The investigators used propensity score matching to compare patients with and without maintenance dialysis using a 1:5 ratio. In contrast to other reported data, there was no difference in inpatient mortality between the two groups (9% vs. 12%). Furthermore, patients on maintenance dialysis seemed to have a lower risk for ICU admission (9% versus 21%) despite having higher inflammatory markers and more comorbid conditions. Regardless of the limitations in using propensity score matching, this is the first study to include a comparison group to contrast differences among patients receiving maintenance dialysis and those who do not.

On the other hand, patients receiving chronic PD have a much lower risk of SARS-CoV-2 infection given the limited need for visits, now more than ever restricted thanks to the implementation of new technologies which allow for telehealth to be a feasible option. Among 818 patients on chronic PD who were followed from January to April in Wuhan, China, only 8 were diagnosed with COVID-19 [86].

Now more than ever, it is imperative that nephrologists advocate for home-based dialysis in order to reduce SARS-CoV-2 exposure. Knowing that there are restrictions to candidature for home dialysis modalities, it is mandated for dialysis facilities to promote the safety of those who choose or already receive in-center hemodialysis. Table 3 summarizes the critical points in mitigating the risk of COVID-19 for dialysis facilities based on the recommendations from several experts [87,88,89].

### 5.3. COVID-19 in Patients with Pre-Existing Glomerulonephritis

The effects SARS-CoV-2 infection has on individuals with immune-mediated glomerulonephritis (GN) were studied by Waldman et al. in the IRoc-GN international registry [90]. In this study, 40 patients with biopsy-proven GN and COVID-19 were compared to 80 controls without GN who were hospitalized with COVID-19. The most common GN in the study was lupus nephritis, followed by focal segmental glomerulosclerosis. Age, sex, and other comorbidities were similar between both groups except for obesity, which was more prevalent in the GN group, though the BMI difference was not statistically different. Mortality among patients with GN was 15% compared to 5% in the control group. AKI was also more common among the GN group when compared to the control (38% vs. 14%), but there were no significant differences in the need for KRT. Twenty out of the forty patients with GN were actively receiving immunosuppression; while calcineurin inhibitors and anti-metabolites were discontinued, corticosteroids were kept in most of the patients. The authors did not find an increased risk of mortality or AKI related to immunosuppression.

Recent guidelines were published by the Immunology Working Group of the European Renal Association—European Dialysis and Transplant Association (ERA-EDTA) for the management of patients with immune-mediated kidney disease during the SARS-CoV-2 pandemic. Among other recommendations, the group suggests to consider stopping or at least reducing the dose of antimetabolites, and postponing infusion of biological drugs in patients with COVID-19. Steroids should not be discontinued abruptly without hydrocortisone replacement [91]. 

### 5.4. COVID-19 in Kidney Transplant Recipients

Kidney transplant recipients may be at particular risk for adverse outcomes from COVID-19 given the use of chronic immunosuppression along with the comorbidities which may be present in this population. The Montefiore Medical Center in New York City was the first to share their experience on 36 kidney transplant recipients diagnosed with COVID-19 [92]. The authors described 10 deaths out of 36 patients (28%). In another retrospective study among 132 kidney transplant recipients positive for COVID-19 at medical centers in The Bronx, New York City, the overall mortality in this group was 20.5%. Interestingly, higher serum IL-6 was associated with increased mortality [52]. The TANGO international transplant consortium performed a retrospective cohort study including 144 transplant recipients admitted with COVID-19 to 12 different centers in the US and Europe [51]. The case fatality rate for this group of patients was 32%. Age above 60 years and higher levels of IL-6 and procalcitonin were associated with increased mortality. Characteristics such as race, comorbidities, selection of induction therapy, maintenance immunosuppression, or use of renin angiotensin system inhibitors were not significantly different among survivors and non survivors.

In addition to augmented case fatality rates, several reports have also noted the overall increase in major adverse outcomes such as admission to the ICU and need for mechanical ventilation. A systematic review on 12 studies and 204 kidney transplant recipients diagnosed with COVID-19 reported that 34% of the patients in the review were admitted to the ICU, while 19.7% required mechanical ventilation [93]. 

The largest dataset looking at the outcomes of kidney transplant recipients with COVID-19 comes from the European Renal Association COVID-19 Database (ERACODA). Their study enrolled 305 kidney transplant patients with COVID-19, 89% of whom required hospital admission. The 28-day probability of death within this group was 21.3%. In this study, the ICU admission rate was 21%, and 18% of the total patients required ventilatory support [94]. 

## 6. Kidney Replacement Therapy and COVID-19

The high incidence of CoV-AKI in the US imposed a significant burden on nephrology departments and ancillary staff. The challenge of providing adequate KRT to a large number of patients in the absence of adequate personal protective equipment (PPE), trained personnel, and dialysis equipment (machines, dialysis solutions, etc.) prompted some institutions to make changes to their protocols and to adapt to this challenging scenario.

### 6.1. Selection of KRT: Which Modality to Use and When to Start

Given the lack of strong data on how to treat CoV-AKI, management strategies have been extrapolated from non-COVID AKI studies. Clinical trials such as the IDEAL-ICU [95], and STARRT-AKI [96], showed that early initiation of KRT does not have a significant impact on mortality reduction or kidney recovery prognosis when compared to standard initiation of KRT based on conventional indications. Although none of these trials were conducted in patients with COVID-19 AKI, it is reasonable to think that similar principles apply to CoV-AKI. However, to date, there are no available data comparing early vs. late initiation of replacement therapies in COVID-19 patients.

The same uncertainty applies to the selection of the preferred modality of kidney replacement therapy. In critically ill patients, there is no significant difference in patients’ outcomes between continuous kidney replacement therapies versus intermittent kidney replacement therapy. Therefore, different modalities are chosen based on institution availability and patient tolerability. Equally to non-CoV-AKI cases, continuous kidney replacement therapies are the modalities of choice in patients with hemodynamic instability who are not able to tolerate intermittent hemodialysis (iHD). No head-to-head trial has been made comparing the different modalities of continuous kidney replacement therapy (CKRT) in CoV-AKI.

There are no solid data to suggest that removal of pro-inflammatory cytokines through convection will improve treatment outcomes or increase survival. There are only small case reports and case series to support the use of extracorporeal therapies or highly adsorptive filters to remove pro-inflammatory molecules. The rationale of removing pro-inflammatory molecules by ultrafiltration seems reasonable, but further studies will be needed in order to assess the potential benefits of these therapies.

Acute PD is a reasonable option to offer continuous or intermittent kidney replacement therapy as an alternative to hemodialysis or hemofiltration. Peritoneal dialysis is rarely used as an option for hospitalized patients with AKI [97]. However, given the shortage of hemodialysis resources, many centers have adopted the use of this modality. El Shamy and collaborators reported their findings in a cohort of 21 patients who were started on peritoneal dialysis for COVID-19-related AKI in a major tertiary care center in New York City [97]. Peritoneal dialysis was successful in providing clearance and ultrafiltration for patients needing KRT during the peak of the pandemic. The most common complication was drainage issues, which were experienced in 19% of patients, while peritonitis happened in only 5% of cases. Another single-center study at Montefiore Medical Center in The Bronx, New York also showed that acute PD is a feasible strategy for patients needing KRT as a consequence of CoV-AKI [98]. The authors of this trial described their experience in 30 patients who were started on PD indicated for CoV-AKI during the first peak of the pandemic in New York City. Out of these patients, 37% received PD as a supplement for other modalities of KRT. Most of the patients (60%) initiated PD in the ICU. The average prescribed PD after 72 h dose was 2-L fill volume, 2-h dwells, and 8 exchanges, conferring a weekly Kt/V of 3.24.

### 6.2. Intrinsic Challenges of KRT in COVID-19 AKI

In the face of shortage of KRT machines and human personnel capable of providing therapy, institutions had to make adjustments to standard treatment practice. Higher dialysate flow rates were used in continuous kidney replacement therapy modalities when treatment time was decreased, and additional extension tubing was used in order to place dialysis and CKRT machines outside patient rooms in order to minimize exposure to COVID-19 for doctors and nurses. Isolated slow continuous ultrafiltration sessions were implemented in between treatments for volume management if diuretics were not effective [99,100]. Similarly, iHD treatment time and frequency were decreased if volume status and metabolic derangements would permit, with potassium binders such as sodium zirconium utilized more readily to control hyperkalemia [101].

### 6.3. Anticoagulation

A particular complication noted in critically ill patients with COVID-19 ARDS is frequent vascular access and dialysis filter clotting. An early retrospective study from Wuhan alluded to the high mortality rate in COVID-19 patients who had abnormal coagulation parameters, compared to those with normal parameters [102]. In a cohort of 150 patients with COVID-19 and ARDS, 97% of those who required KRT had issues with circuit clotting [103]. To conserve resources and prolong CKRT filter life, anticoagulation strategies have been put in place. A retrospective cohort study compared anticoagulation alternatives in 23 critically ill patients who required KRT for CoV-AKI, and who received either continuous veno-venous hemodialysis (CVVHD) or sustained low-efficiency daily dialysis (SLEDD) [104]. Outcomes were reported as mean hours of treatment. For patients on CVVHD, the investigators found that the use of citrate prolonged treatment duration significantly by 24.4 h (*p* = 0.001). Patients receiving SLEDD seemed to benefit from low-molecular-weight heparin (LMWH) over unfractionated heparin or the direct thrombin inhibitor Argatroban (mean dialysis time 11.8 h vs. 8.1 h and 8 h).

Another retrospective study conducted in New York City took a different approach by looking at the life of 502 CKRT circuits on different anticoagulation regimens [105]. Clotting occurred in 35% of the circuits with anticoagulation compared to 52% in those without. The hazard ratio for circuit clotting was higher among citrate users (0.92) and lowest for combination of citrate plus Argatroban (0.21). This study reports data that differ from previous reports in regard to the efficacy of citrate anticoagulation. The group used the combination of citrate plus Argatroban for very high-risk individuals who had already developed thromboembolism. Although efficacious in preventing circuit clotting, the potential risks of combining these two drugs should not be overlooked.

Further studies need to be conducted in order to assess for optimal strategies in anticoagulation for patients with CoV-AKI receiving KRT. Despite disparities in outcomes among individual strategies, it is an undisputed fact that circuits without anticoagulation tend to perform poorly in patients with CoV-AKI when compared to anticoagulated systems. Anticoagulation options for patients on KRT are summarized in Table 4 [106,107,108]. The bleeding risk of each individual patient needs to be considered prior and during the implementation of any of these strategies given that anticoagulation, especially when systemic, may increase the propensity for bleeding. A case series from Italy, for example, described the case of seven patients admitted to the hospital with COVID-19 who presented spontaneous ilio-psoas hematoma as a consequence of either prophylactic or therapeutic anticoagulation with LMWH [109]. None of these patients were receiving KRT. 

## 7. Conclusions

Kidney injury is a common extra pulmonary finding in patients with COVID-19. Though further research is still needed to understand the mechanisms of injury, it is likely that more than one mechanism is implicated. Despite the variability of reports on the incidence of CoV-AKI, we have to expect a high influx of patients and high demands that may overwhelm inpatient nephrology services. As such, it is imperative that centers prepare to match this demand by anticipating the need for human and non-human resources (dialysis machines, filters, dialysate, etc.), and by developing strategies such as the creation of urgent peritoneal dialysis programs to assist hemodialysis services. Physicians also need to be aware of the special challenges in treating COVID-19-related AKI, especially in terms of KRT. There is hope that most of the survivors will have at least partial recovery of kidney function, though we should be preparing for a raise in the incidence of CKD and ESKD.

## Figures and Tables

**Table 1:** Incidence and associated mortality in COVID-19-related acute kidney injury from large series across the world.

	Author	Country	*n* =	ICU (%) ^+^	AKI (%)	KRT (%)	Overall Mortality (%)	Mortality in AKI (%)
1	Cheng et al [21]	China	1400	10%	7%	15%	14%	71%
2	Guan et al [23]	China	1099	5%	0.5%	N/A	1.4%	N/A
3	Xia et al [26]	China	81	100%	51%	9%	74%	82%
4	Yang et al [25]	China	52	100%	23%	5%	61%	N/A
5	Richardson et al [27]	USA	5700	14%	22%	3%	21%	N/A
6	Hirsch et al [9]	USA	5449	26%	37%	14%	16%	35%
7	Fisher et al [36]	USA	3345	4%	57%	5%	9%	34%
8	Chan et al [37]	USA	3235	24%	46%	19%	8%	50%
9	Gupta et al [30]	USA	3099	100%	54%	20%	N/A	55%
10	Argenziano et al [38]	USA	1000	23%	33%	14%	21%	N/A
11	Mohamed et al [35]	USA	575	30%	28%	15%	N/A	50%
12	Diana et al [31]	South Africa	1102	14%	40%	9%	21%	49%^++^
13	El Aidaoui et al [32]	Morocco	134	34%	6%	18%	10.4%	N/A

ICU: intensive care unit, AKI: acute kidney injury, KRT: kidney replacement therapy, N/A: Not Available. + All percentages have been rounded up to the most proximal integer. ++ AKI mortality in this study refers to mortality for AKI Stage 3.

**Table 2:** Common histopathology findings in CoV-AKI biopsy or autopsy series. Percentage of clinical findings is based on number of available data and not on the total of patients in the study. Kidney tropism was determined based on the author’s interpretation, presence of definitive viral like particles in electron microscopy (EM), or molecular evidence of the virus by either immunofluorescence (IF) staining or in situ hybridization (ISH).

	Author	*n*	Sample Collection	Clinical Findings	Histopathology Findings (*n*)
**1**	Santoriello et al [42]	42	Postmortem	AKI: 94% Proteinuria: 79%	ATI: 19 Fibrin Thrombi: 6 CG: 1 Kidney Tropism: Not detected
**2**	Su et al [44]	26	Postmortem	AKI: 35% Proteinuria: 26%	ATI: 26 Fibrin Thrombi: 3 Kidney Tropism: 8
3	Nasr et al [40]	13	Native Kidney Biopsy	AKI: 100% Proteinuria: 100% CKD: 44%	Diffuse ATI: 11 CG: 8 Membranous Nephropathy: 1 Kidney Tropism: ISH performed for one patient only, found to be negative.
4	Kudose et al [41]	17	Native Kidney Biopsy: 14 Kidney allograft: 3	AKI: 88% Nephrotic Range Proteinuria: 53% Median SCr: 5.7 mg/dL	CG: 5 Minimal Change Disease: 1 Membranous nephropathy: 2 Lupus Nephritis: 1 Anti-Glomerular Basement Membrane: 1 T cell-mediated rejection: 3 APOL1 Genotyping: 3 Kidney Tropism: Not detected
5	Akilesh et al [43]	17	Native Kidney Biopsy: 14 Kidney allograft: 3	AKI: 88% Nephrotic Range Proteinuria: 65%	CG: 5 Minimal Change Disease: 1 Thrombotic Microangiopathy: 5 Post-infectious Glomerulonephritis: 1 Antibody-Mediated Rejection: 2
6	Wu et al [45]	6	Native Kidney Biopsy	All patients were Black, had AKI and proteinuria. Nephrotic range proteinuria confirmed in 5/6 cases by UPCR.	CG: 6 APOL 1 high-risk genotype: 6 Kidney Tropism: Not detected

ATI: acute tubular injury, CG: collapsing glomerulopathy, APOL1: apolipoprotein L1, UPCR: urine protein to creatinine ratio, CKD: chronic kidney disease.

**Table 3:** Strategies aiming to mitigate spread of SARS-CoV2 in dialysis units.

**Isolation of Sick Patients**
Designate isolation shifts
If possible, use separate rooms for confirmed cases or cases under investigation
**Screening**
Screening personnel at a single entry to the facility
Screen for symptoms and exposure
Check body temperature prior to the start of dialysis
Medical evaluation for patients with positive screenings and test when appropriate
**Patient Education**
Encourage Vaccination
Provide information about vaccine alternatives
Provide information about vaccination centers
Assist with enrollment
Proper use of Face masks
Appropriate hand wash
Patients should be encouraged to wash their dialysis access arm
Use of individual transport to and from dialysis facilities
Patients should alert beforehand if new symptoms appear, or if they are exposed to patients with COVID-19
**Disinfection**
Disinfect dialysis machines, chair, dialysis stations, and all equipment (BP cuffs, stethoscopes, etc.)
**Communication with Health Department**
Detect cases and communicate to local health authorities
**Healthcare Teams**
Provide appropriate personal protective equipment including face shields, isolation gowns, and N-95 fitted masks for all dialysis staff.

**Table 4:** Anticoagulation Strategies for kidney replacement therapy (KRT) in CoV-AKI. Anticoagulation strategies in KRT adopted from recommendations given by a panel of experts from the American Society of Nephrology (ASN) in their webinar on April 2020, along recommendations from 2012 Kidney Disease Improving Global Outcomes (KDIGO) guidelines.

Anticoagulant	Dose	Remarks
Pre-filter Unfractionated Heparin	Loading dose: 2000–5000 units. Maintenance: 10–15 units/kg/hr Check PTT: 2–4 h after initiation, Target 5 sec increase.	Anticoagulation is intended for the circuit, not for the patient Higher risk of circuit clotting for high-risk patients
Systemic Unfractionated Heparin	Loading dose: 50–80 units/kg Maintenance: Continuous drip at 18–20 units/kg/h Target PTT: 80–100	Protocols may vary Higher risk of bleeding when comparted to pre-filter heparin Increased risk of HIT and heparin resistance Short half-life
Systemic Low-Molecular-Weight Heparin	Dose may be variable, and single pre dialysis dose may be sufficient.	Risk of accumulation in kidney failure Monitoring requires anti-Factor Xa Reduce risk of HIT
Regional Citrate Anticoagulation	No universal protocol.	Requires institutional commitment Better safety profile than heparin Risk for overdose, metabolic acidosis, and hypocalcemia Increased monitoring of iCa and titration of CaCl.
Argatroban	0.5 mcg/kg/min if normal liver function 0.2–0.25 mcg/kg/min in patients with liver dysfunction Target PTT: two times the normal value and titrate based on institutional protocol	Variable institutional protocols Dose differently for those with and without liver dysfunction Use if HIT

PTT: partial thromboplastin time, HIT: heparin induced thrombocytopenia, iCa: ionized calcium, CaCl: calcium chloride.

## Data Availability

Not applicable.
